# Endometrial and follicular development following stair-step and traditional protocols in women with polycystic ovary syndrome: An RCT

**DOI:** 10.18502/ijrm.v19i6.9375

**Published:** 2021-07-27

**Authors:** Sholeh Shahgheibi, Fariba Seyedoshohadaei, Danial Khezri, Solmaz Ghasemi

**Affiliations:** ^1^Department of Obstetrics and Gynecology, Faculty of Medicine, Kurdistan University of Medical Sciences, Sanandaj, Iran.; ^2^Student Research Committee, Kurdistan University of Medical Sciences, Sanandaj, Iran.; ^3^Besat Hospital, Kurdistan University of Medical Sciences, Sanandaj, Iran.

**Keywords:** Polycystic ovary syndrome, Clomiphene, Infertility, Ovulation induction.

## Abstract

**Background:**

Various strategies have been proposed for polycystic ovary syndrome (PCOS) treatment.

**Objective:**

To investigate and compare the number and size of ovarian follicles, endometrial thickness, and ovulation rate by traditional protocol (TP) and stair-step protocol (SSP).

**Materials and Methods:**

Sixty infertile PCOS women were allocated into two groups (SSP = 30 and control TP = 30) between May and October 2019 in the Besat Hospital, Sanandaj, Iran. In the SSP group, the infertile women were treated with 50 mg/daily clomiphene citrate (CC) for five days, while the nonresponsive women were prescribed 100 mg daily CC for five days in the same cycle. The maximum dose (150 mg) was administered until ovulation occurred. In the control group, in non-ovulatory cases, the dose was increased in the next cycle. Ultrasound was used to detect ovulation.

**Results:**

Endometrial thickness changes with various doses of CC were significantly different in the TP. The comparison of both protocols showed a significant difference in endometrial thickness only at 50 mg CC. The number of follicles in the left ovary was significantly different in both protocols at 150-mg CC. The size of ovarian follicles in the left ovary was significantly different between the two protocols at 100-mg CC. The ovulation rate was significantly different in the SSP at 100- and 150-mg doses of CC. Moreover, 86% of ovulation occurred at 100-mg CC in the SSP, while this rate was 73% in the TP.

**Conclusion:**

The most appropriate dose for ovulation in patients with PCOS is 100 mg CC.

## 1. Introduction

Anovulation causes about 40% infertility in women, and many infertile patients suffer from polycystic ovary syndrome (PCOS) (1). PCOS is one of the most prevalent endocrine disorders in women of childbearing age. It occurs in about 4–12% of cases (2) and is characterized by irregular menstruation following periods of normal menstruation, hyperandrogenism with varying degrees of hirsutism, hair loss, and PCOS diagnosed by ultrasound (3).

PCOS has an unknown etiology. However, Yen presented a hypothesis based on two principles: hyperandrogenism and insulin resistance. Imbalanced secretion of luteinizing hormone (LH) and follicle-stimulating hormone at the surface of the pituitary gland stimulates the secretion of endogenous LH. Imbalanced secretion of LH and follicle-stimulating hormone at the surface of the pituitary gland stimulates the secretion of endogenous LH. LH highly stimulates the production of androgen in the ovaries, and androgen converts to estrogen during the aromatization process in granulosa cells. Insulin and LH can directly induce the production of steroid hormones in ovaries, especially ovarian androgen. Moreover, insulin reduces the production of sex hormone-binding globulin in the liver, thereby increasing the free androgen in blood. Therefore, these two pathways stimulate the single ovarian cells and increase the production of androgens from ovaries, which in turn results in follicular dysfunction (folliculogenesis), abnormal menstruation cycle, and chronic oligo-anovulation (4).

Use of clomiphene citrate (CC) is a common first-line treatment in patients with PCOS and anovulation, to which 75–80% of patients respond (5). Although CC successfully induces ovulation, there is a significant difference between ovulation and pregnancy rate, which is partly due to the peripheral anti-estrogenic effects of CC on the endometrial surface and cervical mucosa. In addition, the long half-life of CC causes high LH secretion and a negative effect on granulosa cells and oocytes (6).

A new protocol for the treatment of PCOS is stair-step protocol (SSP), in which a daily increase of CC occurs in a cycle. An important point in this protocol is regular monitoring of ovary activity during stimulation by ultrasound. A potential advantage of this protocol is the absence of waiting time until the next menstruation to increase the drug dose. The negative effects of cumulative doses of CC in a cycle on the endometrium and its systemic side effects on the patient are the drawbacks of this protocol (7). The induction of ovulation usually starts with 50 mg/daily CC for five days in patients with PCOS. Of them, 52% ovulate with 50-mg CC, 22% with 100-mg CC, 12% with 150-mg CC, 7% with 200-mg CC, and 5% with 250-mg CC (8).

This study investigated the ovarian follicular response to different CC doses in traditional protocol (TP) and SSP to find out whether changing CC concentrations would induce ovarian follicular growth. Endometrial thickness, number and size of follicles in each ovary at different doses of CC, and ovulation rate in each protocol were studied and compared. Finally, both protocols concerning the above variables were compared.

## 2. Materials and Methods

This single-blind clinical trial was conducted on 85 women with PCOS referred to the gynecology and infertility center of Besat Hospital, Sanandaj, Iran between May and October 2019. Finally, 60 women with PCOS were included in the study. Patients were randomly divided into intervention and control groups based on randomized block method. PCOS was diagnosed based on the Rotterdam criteria, that is, two out of the following three criteria: irregular menstrual cycle (oligo-amenorrhea), clinical or biochemical evidence of hyperandrogenism, or evidence of PCOS by ultrasound (9). Infertility due to PCOS resistant to treatment and willingness to participate in the study was also an inclusion criteria. The exclusion criteria of the study were age over 40 yr. The demographic information of patients was obtained from their medical history and body mass index (kg/m${}^{2}$).

A total of 60 women with PCOS who had not responded to treatment with 50-mg/daily CC for five days were allocated to the control (TP) and intervention (SSP) groups. In the SSP group (n = 30), 50-mg/daily CC was administered for five days after the onset of the cycle and the follicular response was studied eight days after the vaginal ultrasound. If the size of the follicles is less than 12 mm, 100 mg of clomiphene citrate is prescribed daily for another 5 days and an ultrasound is performed on the twentieth day of menstruation. If the size of the follicles is less than 12 mm, 150 mg of CC daily for another 5 days. Citrate is prescribed and an ultrasound is performed on the 25${}^{\mathrm{th}}$ day of menstruation. Intervention group 2: From the third day of menstruation, 50 mg of clomiphene is prescribed daily for 5 days, and on the 14${}^{\mathrm{th}}$ day of menstruation, ultrasound is performed, and if ovulation is not induced, in the next month, the treatment is repeated with 100 mg. If ovulation is not induced, treatment with 150 mg is repeated in the next month (third month).

**Figure 1:**
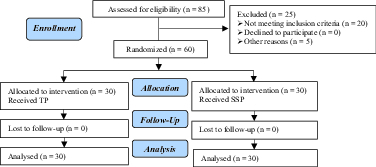
Consort diagram of the selection and treatment of the patients.

In both protocols, the onset of the cycle was defined as the first day of treatment. Lack of response to treatment was regarded as unsuccessful ovulation (Figure 1).

This study evaluated the ovarian status by comparing the TP and SSP in terms of the number and size of follicles, ovulation rate, and changes in endometrial thickness by increasing the CC dose in each protocol.

### Ethical considerations

All participants gave informed written consent prior to the study. This study was approved by the ethics committee of the Kurdistan University of Medical Sciences (IR.MUK.REC.1397.268), and is also registered with the Iranian Registry of Clinical Trials.

### Statistical analysis

Kolmogorov–Smirnov test was used to evaluate the normal distribution of data and the results showed that the distribution of SSP and TP data is not normal, so the non-parametric U Mann–Whitney test was used to analyze them. Other variables were compared using *t* test and Chi-square. Doses of 100 and 150 mg CC were considered as two protocols and the variables were compared with these two protocols. Pearson correlation coefficient and multiple regression models were used to examine the relationship between variables. Data were analyzed using SPSS statistical package version 21. P-values < 0.05 were considered as statistically significant.

## 3. Results

A total of 60 women underwent ovulation induction by two protocols. The mean age of SSP and TP groups were 26.20 ± 1.52 and 28.13 ± 1.92 yr, respectively (Table I). A total of 23 patients (76.7%) were ovulated by the SSP and 21 (70%) by the TP. A comparison of endometrial thickness in the TP in three doses of CC showed a significant difference in endometrial thickness following a CC dose increase. Significant differences were also seen in the endometrial thickness in the SSP with the 50- and 100-mg CC (p < 0.001), but no significant difference was found between the 50- and 100-mg doses in terms of endometrial thickness (p = 0.02). A comparison of endometrial thickness in both protocols indicated a significant difference in endometrial thickness only at 50-mg CC in both protocols (p = 0.03) (Table II). The results showed that in the SSP group, 17 women and in the TP group, 16 women needed a higher dose of clomiphene (Table II).

The findings of this study showed a significant difference between the number of follicles in the right ovary and the number of follicles in the left ovary in the SSP and TP groups for 150-mg CC (Table III).

Based on our findings, there was a significant difference between the SSP and TP groups for 150-mg CC regarding the NFLO (Table III).

Tables III and IV present the comparison of the number and size of ovarian follicles and the ovulation rate in each protocol and a comparison of both protocols. The findings showed 86% ovulation in the SSP and 73% in the TP with 100-mg CC treatment (Table IV).

**Table 1:** Comparison of demographic characteristics of the SSP and TP groups


	**SSP**	**TP**	**P-value** ${}^{†}$
Age (yr)${}^{*}$	26.20 ± 1.52	28.13 ± 1.92	0.11
BMI (kg/m ${}^{2}$)${}^{*}$	28.74 ± 1.22	28.86 ± 1.38	0.89
The duration of marriage (months)${}^{*}$	76 ± 1.34	84 ± 1.80	0.59
The duration of infertility (months)${}^{*}$	28 ± 0.74	30 ± 0.86	0.36
${}^{*}$Data presented as Mean ± SD. ${}^{†}$ *t* test, BMI: Body mass index, SSP: Stair-step protocol, TP: Traditional protocol			

**Table 2:** Endometrial thickness in the different dose of CC in the SSP and TP groups


**Dose (mg)**	**CC**				**P-value${}^{†}$**
	**N**	**SSP**	**N**	**TP**	
**50**	30	6.97 ± 1.20	30	6.18 ± 1.25	0.03
**100**	29	8.32 ± 2.37	30	8.30 ± 2.96	0.098
**150**	17	9.27 ± 2.49	16	8.12 ± 2.78	0.20
Data presented as Mean ± SD. **${}^{†}$**Mann–Whitney Test, CC: Clomiphene citrate, SSP: Stair-step protocol, TP: Traditional protocol					

**Table 3:** The effect of different doses of CC on the mean size and number of follicles per ovary in the two protocols


			**100-mg CC**			**150-mg CC**		
			**N**	**Mean**	**P-value ${}^{†}$**	**N**	**Mean**	**P-value ${}^{†}$**
**Number of follicle**								
	**NFLO**							
	**TP**	30	0.33	0.56	30	0.10	0.04
	**SSP**	30	0.43	30	0.37	
	**NFRO **							
	**TP**	30	0.73	0.86	30	0.30	0.51
	**SSP**	30	0.77	30	0.43	
**Size of follicle**								
	**SFLO**							
	**TP**	10	16.70	0.32	3	17.67	0.12
	**SSP**	13	15.38	11	15.18	
	**SFRO**							
	**TP**	22	16.55	0.17	9	18.78	0.32
	**SSP**	23	16.83	13	17.46	
Data presented as Mean ± SD, ${}^{†}$Mann–Whitney Test, N: Number of patients, TP: Traditional protocol, SSP: Stair-step protocol, NFRO: Number of follicle in right ovary, NFLO: Number of follicle in left ovary, SFRO: Size of follicle in right ovary, SFLO: Size of follicle in left ovary, CC: Clomiphene citrate								

**Table 4:** Comparison of the effect of CC doses on study variables in the two protocols


**Variables**	**Dose**	**TP**	**P-value ${}^{†}$**	**SSP**	**P-value ${}^{†}$**
**Endometrial thickness**	50–100	6.18–8.30	0.01	6.97–8.32	0.00
	100–150	8.30–8.12	0.00	8.32–9.27	0.20
**NFRO**	100–150	0.73–0.3	0.03	0.77–0.43	0.10
**NFLO**	100–150	0.33–0.10	0.45	0.43–0.37	0.24
**SFRO**	100–150	16.55–18.78	0.34	16.83–17.46	0.28
**SFLO**	100–150	16.70–17.67	–	15.38–15.18	0.32
**Ovulation rate**	100–150	43.47–72.83	0.18	18.67–23.15	0.04
Data presented as Mean ± SD, ${}^{†}$Mann–Whitney Test, NFRO: Number of follicle in right ovary, NFLO: Number of follicle in left ovary, SFRO: Size of follicle in right ovary, SFLO: Size of follicle in left ovary, CC: Clomiphene citrate, SSP: Stair-step protocol, TP: Traditional protocol					

## 4. Discussion

In this study, CC was administered by SSP and TP in women with PCOS. To the best of our knowledge, few studies have been done to compare the SSP and TP, so any findings in this regard can be helpful.

In this randomized clinical trial study, we evaluated the number and size of ovarian follicles, endometrial thickness, and ovulation rate by clomiphene SSP and TP in women with PCOS. In our study, the mean age of SSP and TP groups were 26.20 ± 1.52 and 28.13 ± 1.92 yr, respectively. In a study by Jones and colleagues on 109 women with PCOS, the mean age in the SSP and TP groups were 32.6 ± 1.5 and 33.1 ± 1.2 yr (10) and in a study by Al Ghazali to evaluate and compare the efficacy of clomiphene SSP and TP to induce ovulation in women with PCOS, the mean age of SSP and TP groups were reported as 27.65 ± 5.63 and 28.58 ± 5.97 yr, respectively (11). In two other previous studies also, the mean age were reported as 26.17 ± 3.19 and 26.87 ± 3.87 (12) and 23.1 ± 3.7 and 24.9 ± 3.5 yr, respectively (7).

The results of this study showed that the endometrial thickness in the TP group in three doses of CC (50, 100, 150 mg) increased following a CC dose increase. However, significant differences in endometrial thickness occurred in the SSP with 50- and 100-mg CC, but no significant difference was found between 50- and 100-mg doses. The endometrial thickness in both SSP and TP protocols indicated a significant difference only at 50-mg CC. Previous studies reported no significant differences in SSP compared to TP regarding endometrial thickness (7, 11, 12, 13). One of the most important drawbacks of CC is its antiestrogenic effect on the normal growth of endometrium so that it inhibits the normal endometrial growth and uterine thickness. Increasing the CC dose induces more negative effects on the endometrium (14). There are contradictory reports about the effect of CC on endometrial growth. Homburg reported that a lack of endometrial growth was not associated with the dose and duration of CC consumption. CC was found to merely play an underlying but an idiosyncratic role in this regard (15). Uterine arterial blood flow increases from the follicular phase to ovulation in normal cycles. However, this increase is not recognizable in ovulation-induction phases caused by CC. Further, CC possibly inhibits endometrial changes by negatively affecting the endometrial receptors (16).

Our findings showed that the number and size of ovarian follicles are affected by different doses of CC in two protocols. There was a significant difference between the number of follicles in the left ovary in SSP and TP groups for 150-mg CC, but in 50- and 100-mg CC, no significant difference was observed. Although there was a difference in the size of ovarian follicles of the SSP and TP group, the difference was not statistically insignificant. Al Ghazali (11) reported the highest number of follicles and the largest size of follicles in the SSP group. In another study also found a statically significant difference between the two SSP and TP groups regarding the number of follicles, wherein the highest number of follicles was recorded by SSP versus the TP (17). CC increases the secretion of follicle stimulating hormone and LH by exerting its estrogenic effects on the central nervous system, which in turn has stimulatory effects on gonadotropin and ovaries and overcomes ovarian disorders. This function elevates the number of ovulatory follicles. Increasing the number of eggs, the chance of pregnancy is increased (18). There are two theories about the number of induced follicles, some have reported more in the TP (19, 12), and some in the SSP (10, 20). Some studies have shown a positive relationship between follicular growth and increased CC dose (18–21). However, treatment with 50–mg/daily CC for five days usually induces the growth of 1–3 follicles (10).

The results of the present study showed no significant difference between the 100- and 150-mg CC for ovulation rate in the TP group. Therefore, it can be inferred that increasing the CC dose from 100 to 150 mg has no effect on the ovulation rate in the TP group, while there was a statistically significant difference between the 100- and 150-mg CC doses in the SSP group regarding the ovulation rate. Moreover, a comparison of both protocols indicated a significant difference between the 100- and 150-mg doses of CC regarding the ovulation rate. 100 mg CC was the most effective dose for ovulation in both groups (12), which was similar to our finding. 64% of ovulation occurred in SSP and 22% in TP at a dose of 100 mg (13). Higher ovulation rate in SSP group compared to TP (7). Ovulation rate was 43% per 100 mg with SSP compared with 25.3% with TP and concluded that clomiphene SSP improved ovulation rate in clomiphene resistant women (11). There are reports of higher ovulation rates in the SSP group compared to the TP group (17). Furthermore, in a study by Horowitz and Weissmani, it was concluded that for women with an ovulatory PCOS, the CC-SS protocol is associated with increased ovulation rate at higher doses when compared with the TP (22). Our findings were similar to the previous studies (19–22).

## 5. Conclusion

Based on our findings, it can be concluded that 100-mg/daily CC is the most appropriate dose for ovulation induction in women with PCOS.

##  Conflict of Interest

The authors declare that they have no competing interests.
